# Maturation of Corticospinal Tracts in Children With Hemiplegic Cerebral Palsy Assessed by Diffusion Tensor Imaging and Transcranial Magnetic Stimulation

**DOI:** 10.3389/fnhum.2019.00254

**Published:** 2019-07-24

**Authors:** Christos Papadelis, Harper Kaye, Benjamin Shore, Brian Snyder, Patricia Ellen Grant, Alexander Rotenberg

**Affiliations:** ^1^Laboratory of Children’s Brain Dynamics, Division of Newborn Medicine, Boston Children’s Hospital, Harvard Medical School, Boston, MA, United States; ^2^Fetal-Neonatal Neuroimaging and Developmental Science Center, Division of Newborn Medicine, Boston Children’s Hospital, Harvard Medical School, Boston, MA, United States; ^3^Neuromodulation Program, Division of Epilepsy and Clinical Neurophysiology, Department of Neurology, Boston Children’s Hospital, Boston, MA, United States; ^4^F.M. Kirby Neurobiology Center, Boston Children’s Hospital, Boston, MA, United States; ^5^Department of Orthopedic Surgery, Boston Children’s Hospital, Harvard Medical School, Boston, MA, United States; ^6^Department of Radiology, Boston Children’s Hospital, Harvard Medical School, Boston, MA, United States; ^7^Department of Neurology, Berenson-Allen Center for Noninvasive Brain Stimulation, Division of Cognitive Neurology, Harvard Medical School and Beth Israel Deaconess Medical Center, Boston, MA, United States

**Keywords:** hemiplegic cerebral palsy, corticospinal tracts, development, maturation, transcranial magnetic stimulation

## Abstract

**Aim**: To assess changes in the developmental trajectory of corticospinal tracts (CST) maturation in children with hemiplegic cerebral palsy (HCP).

**Methods**: Neuroimaging data were obtained from 36 children with HCP for both the more affected (MA) and less affected (LA) hemispheres, and, for purposes of direct comparison, between groups, 15 typically developing (TD) children. With diffusion tensor imaging (DTI), we estimated the mean fractional anisotropy (FA), axial diffusivity (AD), mean diffusivity (MD), and radial diffusivity (RD) of the corticospinal tract, parameters indicative of factors including myelination and axon density. Transcranial magnetic stimulation (TMS) was performed as a neurophysiologic measure of corticospinal tract integrity and organization. Resting motor threshold (rMT) was obtained per hemisphere, per patient.

**Results**: We observed a significant AD and MD developmental trajectory, both of which were inversely related to age (decrease in AD and diffusivity corresponding to increased age) in both hemispheres of TD children (*p* < 0.001). This maturation process was absent in both MA and LA hemispheres of children with HCP. Additionally, the TMS-derived previously established rMT developmental trajectory was preserved in the LA hemisphere of children with HCP (*n* = 26; *p* < 0.0001) but this trajectory was absent in the MA hemisphere.

**Conclusions**: Corticospinal tract maturation arrests in both hemispheres of children with HCP, possibly reflecting perinatal disruption of corticospinal tract myelination and axonal integrity.

## Highlights

– Linear age-dependent developmental trajectories of corticospinal tracts diffusion metrics
– Halted bilateral corticospinal tracts imaging metrics maturation in children with HCP
– Preserved resting motor threshold maturational trajectory in the less affected hemisphere of children with HCP
– Absent resting motor threshold maturational trajectory in the more affected hemisphere of children with HCP

## Introduction

Hemiplegic cerebral palsy (HCP) is a common subtype of motor dysfunction, affecting one-third of patients with a clinical cerebral palsy diagnosis (Hagberg et al., 2001). Children with HCP reliably exhibit prominent impairment in skilled voluntary movements. The underlying etiology is a non-progressive lesion located most commonly in the periventricular white matter of the developing fetal or infant brain (Rosenbaum et al., 2007). This lesion impairs the structural integrity of the corticospinal tracts (CST), which are the most important tracts for fine motor skills, and among the first tracts to mature. Despite extensive literature showing microstructural damage in the CST of children with HCP (Scheck et al., 2012), little is known about how the underlying lesion affects the maturation process of these fibers.

CST maturation is a complex process affected by dynamic factors such as synaptic pruning and development (Eyre et al., 2001), myelination (Eyre et al., 1991), changes in axonal diameter and length (Eyre et al., 2002) and organization of pyramidal neuron firing patterns (Chiappa et al., 1991). Several studies have reported measurable CST developmental changes in healthy children using either transcranial magnetic stimulation (TMS) of the motor cortex (Koh and Eyre, 1988; Nezu et al., 1997; Paus et al., 2001) or diffusion tensor imaging (DTI; Lebel and Beaulieu, 2011; Yeo et al., 2014) of the CST. When measured by TMS, CST maturation in healthy children corresponds to a progressive increment of cortical excitability from infancy to adulthood that completes in mid-adolescence (Koh and Eyre, 1988; Nezu et al., 1997; Hameed et al., 2017; Kaye and Rotenberg, 2017). An analogous developmental trajectory is also seen when CST maturation is measured by DTI: a steep increase of the fiber volume and fractional anisotropy (FA) is observed in healthy children until early adolescence and a later gradual increase until adulthood (Lebel and Beaulieu, 2011; Yeo et al., 2014). The mechanistic relationship between these measures seems logical as larger myelinated fiber caliber should correspond to increased excitability. Yet, there are no TMS or DTI studies to examine whether and how normal CST maturation is affected by perinatal white matter injury, as occurs in HCP.

Here, we describe a cross-sectional study investigating the developmental trajectories of TMS and DTI CST metrics as a function of age in children with HCP to test whether perinatal injury arrests normal CST development, and whether such an arrest is confined to the more affected (MA) hemisphere of children with HCP. Specifically, by TMS, we measure the resting motor threshold (rMT), which reflects motor cortex excitability and the developmental stage of CST myelination, as well as the membrane characteristics and synaptic efficacy of the cortical and spinal motor neurons (Garvey et al., 2003). By DTI, we assess the structural integrity of the CST by measuring the FA, axial diffusivity (AD), mean diffusivity (MD), and radial diffusivity (RD), which are parameters indicative of myelination and axon density, among other factors (Grant et al., 2001).

## Materials and Methods

### Participants

Neuroimaging data were obtained from 36 children and adolescents with HCP (age = 11.83 ± 3.79 years; range: 4.1–17.8 years; 17 females) and, for direct comparison, 15 age-matched typically developing (TD) children and adolescents (age = 12.05 ± 3.67 years; range: 7.13–18.02 years; 9 females). The inclusion criteria were: (i) mild to moderate spastic hemiplegia [Gross Motor Function Classification System (GMFCS) level I, II, or III; Manual Abilities Classification Scale (MACS) level I, II, or III]; (ii) sufficient cooperation to participate in a neuroimaging study; (iii) no contradiction for magnetic resonance imaging (MRI); i.e. presence of metallic implants, or pumps; and (iv) no severe intellectual developmental disability. The clinical characteristics of participants with HCP are shown in Table 1. The TD children were recruited from the local community. This study was carried out in accordance with the recommendations of Boston Children’s Hospital (BCH) Internal Review Board (IRB). All subjects gave written informed consent in accordance with the Declaration of Helsinki. The protocol was approved by Boston Children’s Hospital IRB (IRB-P00023570; PI: CP).

**Table 1:** Patient demographics and MRI findings.

ID	Age (Years)	Handedness	Epilepsy	GMFCS	MACS	MA hemisphere	Lesion type
CH 1	4	Left	Y	2	2	Left	Perinatal Stroke
CH 2	5	Right	Y	1	1	Right	Perinatal Stroke
CH 3	6	Left	Y	1	1	Left	Perinatal Stroke
CH 4	7	Right	N	2	1	Right	PV-WMI
CH 5	7	Right	Y	1	1	Left	CE
CH 6	7	Left	N	1	1	Left	Perinatal Stroke
CH 7	8	Right	Y	3	3	Right	Perinatal Stroke
CH 8	8	Left	Y	2	2	Left	Perinatal Stroke
CH 9	10	Right	Y	2	2	Left	PVNH
CH 10	10	Right	Y	2	2	Left	Perinatal Stroke
CH 11	10	AMBI	Y	1	1	Left	CE
CH 12	10	Left	Y	2	3	Left	Perinatal Stroke
CH 13	11	Right	Y	1	1	Left	CE
CH 14	11	Right	N	1	2	Right	Perinatal Stroke
CH 15	11	Right	N	1	1	Left	Parenchymal Atrophy
CH 16	11	Left	Y	1	1	Left	Perinatal Stroke
CH 17	11	Right	Y	2	2	Right	CE
CH 18	11	Left	Y	1	1	Left	CE
CH 19	11	Left	Y	2	3	Right	Perinatal Stroke
CH 20	12	AMBI	Y	1	1	Right	Perinatal Stroke
CH 21	12	Left	Y	1	1	Left	PV-WMI
CH 22	13	Left	Y	2	2	Left	CE
CH 23	13	Left	N	1	1	Left	PV-WMI
CH 24	14	Right	N	1	2	Right	Perinatal Stroke
CH 25	14	Right	N	1	2	Left	Parenchymal Defect
CH 26	15	Right	Y	2	2	Right	CE
CH 27	15	Right	Y	1	1	Right	Perinatal stroke
CH 28	16	Right	Y	1	1	Right	Perinatal stroke
CH 29	16	Right	Y	2	2	Right	CE
CH 30	16	Right	N	1	1	Right	PV Gliosis
CH 31	16	Left	Y	1	1	Left	Perinatal stroke
CH 32	17	Left	N	1	2	Left	CE
CH 33	17	AMBI	Y	1	1	Right	CE
CH 34	17	Right	Y	1	2	Left	CE
CH 35	18	Left	Y	1	2	Left	Perinatal stroke
CH 36	18	Left	N	1	2	Right	CE

*Patient demographics include age in years; handedness; epileptic status; Gross Motor Function Classification System (GMFCS) level; Manual Abilities Classification Scale (MACS) level; the MA hemisphere; Lesion Type. AMBI, ambidextrous; PV-WMI, Periventricular White Matter Injury; PV Gliosis, Periventricular Gliosis; CE, Cystic Encephalomalacia; PVNH, Periventricular Nodular Heterotropia*.

### Image Acquisition

MRI scans were performed in a 3T Magnetom Tim Trio (Siemens Healthcare, Germany). The imaging protocol consisted of structural and diffusion-weighted sequences. The structural sequence was a T1-weighted magnetization-prepared rapid-acquisition gradient-echo acquisition (MPRAGE), which used volumetric echo-planar (EP) imaging navigators for real-time motion correction [voxel size (mm) = 1.0 × 1.0 × 1.0; field of view (FOV) = 19.2–22.0 cm; echo time (TE) = 1.74 ms; repetition time (TR) = 2,520 ms; flip angle = 7°]. The FOV was set to 256 mm and matrix size was 256 [TR = 3,200 ms, TE = 363 ms, Generalized Autocalibrating Partial Parallel Acquisition (GRAPPA) acceleration *R* = 2, echo-spacing of 3.63 ms for a total imaging time of 3:23 min]. The diffusion sequence (prescribed axially) used EP readouts [voxel size (mm) = 2.0 × 2.0 × 2.0; FOV = 11–12.8 cm; TE = 88 ms; TR = 8,320–10,934 ms; flip angle = 90°; 30 gradient diffusion directions at *b* = 1,000 s/mm^2^; 10 acquisitions with *b* = 0 s/mm^2^].

### Identification of More and Less Affected Hemispheres

The MRI scans were reviewed by a pediatric radiologist (PEG). No structural abnormalities were observed in the MRIs of TD children. A unilateral structural abnormality was seen in 31 children with HCP. In five cases, bilateral abnormalities were observed. The MA hemisphere referred to the hemisphere where structural abnormalities were identified or were more prominent compared to the other hemisphere. For all children with HCP, the MA hemisphere was contralateral to the paretic hand. Each child in the TD group was assigned to have a randomly selected hemisphere (Hem1 and Hem2) to balance out possible inherent differences in the functioning of the right and left hemispheres (Pihko et al., 2014).

### DTI Analysis

**Figure 1:**
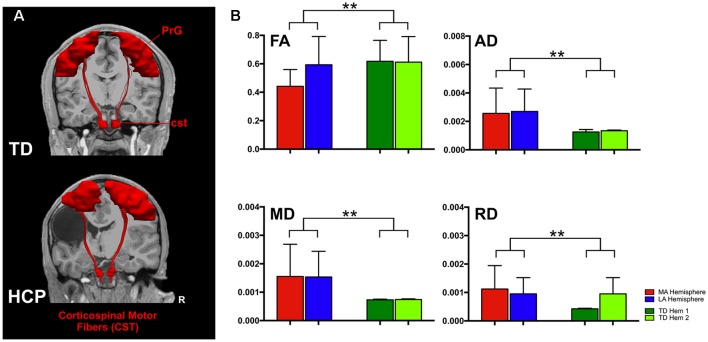
Anatomically defined region of interest (ROI) and corticospinal tracts (CST). **(A)** The ROIs pre-central gyrus (PrG and cst) and their corresponding CST for a typically developing (TD) child (aged 18 years, upper panel) and a child with hemiplegic cerebral palsy (HCP) overlaid on their magnetic resonance imagings (MRIs). **(B)** Error bars (mean ± 95% confidence interval) of diffusion parameters [fractional anisotropy (FA), axial diffusivity (AD), mean diffusivity (MD), and radial diffusivity (RD)] for the CST for both hemispheres of TD children and the less affected (LA) and more affected (MA) hemispheres of children with HCP (**p < 0.001).

From our cohort, DTI data were available for 17 children with HCP (mean: 12.93 ± 3.8 years; range: 6.59–17.80 years; nine females) and all TD children. Diffusion images were first processed to correct for distortions caused by minor eddy currents and simple head motions using FSL tools^1^. Diffusion tensor models were estimated with tractography plugin in the Children’s Research Integration System (ChRIS) using the Fiber Assignment by Continuous Tracking (FACT) method and an angle threshold of 45° with no FA threshold (Mori et al., 1999). The algorithm also generated FA maps, vector maps, and a color-coded direction map. Colors were assigned by direction and orientation of the fibers (blue: superior-inferior, green: antero-posterior, red: left-right). Tracts within these directions were represented with a combination of these three colors. Volumetric segmentation and cortical surface reconstruction of T1-weighted images were performed with Freesurfer^2^. The T1 image was co-registered to the diffusion space using command-line tools from Freesurfer for visualization purposes of the tracts. The generated track file was uploaded into Trackvis^3^ to analyze the diffusion data and create regions of interest (ROIs) for the targeted areas. To track the CST, the ROIs were placed over the pre-central gyrus (PrG) and cst at the brainstem level. The PrG and cst were manually defined using anatomical landmarks of the participant’s MRI along with the color-coded direction maps and a diffusion atlas for reference. Figure 1A presents a 3D representation of the anatomically-defined ROIs and the corresponding CST for a TD and a child with HCP. Mean scalar measures of FA, AD, MD, and RD were derived for each fiber track. Data were analyzed separately for the two hemispheres identified as MA and less affected (LA) for the children with HCP and for both hemispheres of TD children. Only patients with identifiable CST in both hemispheres were considered for further analysis.

### TMS

From our cohort, 26 children with HCP (age = 11.47 ± 3.79 years; range: 4.1–17.8 years; 12 females) underwent motor mapping with TMS. No TD children participated in the TMS motor mapping session. Each participant’s T1-weighted MPRAGE was converted to a 3D head surface and brain reconstruction using Nexstim 4.3 software (Nexstim, Finland), and optimal cortical peel depth was chosen based upon individual cortical anatomy. TMS, coupled with surface electromyography (EMG), was delivered *via* a figure-of-eight coil with frameless stereotaxy and TMS neuronavigation software that allows for continuous visualization of the stimulation coil relative to the patient’s individual brain MRI. Real-time stimulus-locked EMG was recorded from pre-determined target muscles, with one common-ground EMG amplifier (band-pass filter 10–500 Hz, sampling rate 3 KHz per channel). Surface EMG electrodes were placed on the right and the left abductor pollicis brevis (APB) muscles, and a ground electrode on the underside of the right forearm. With single-pulse TMS, stimuli were applied to scalp sites overlying the motor cortex, while muscle activity was monitored in real-time with stimulus-locked EMG. Motor evoked potentials (MEPs) were recorded bilaterally from the APB muscles and a hotspot, corresponding to location that produced peak APB MEP amplitudes were identified per hemisphere. Thereafter, rMT was determined as the minimum stimulation intensity (recorded as electric field strength, V/m) at the APB hotspot that was necessary to elicit a response from the APB, contralateral to the stimulated hemisphere, of 50 μV, on ≥50% of trials. The rMT was determined as percent machine output (MO) and in the corresponding units (V/m) of the induced electric field (e-field; Julkunen et al., 2012). Both rMT determination and motor mapping were performed separately in each hemisphere per child.

### Statistical Analysis

Statistical analysis was performed using GraphPad Prism Software v.7 (GraphPad Prism Software, La Jolla, CA, USA). For the diffusion parameters, we compared the mean FA, AD, MD, and RD of the CST with a mixed 2 (group: HCP, TD) × 2 (hemisphere: LA, MA) analysis of variance (ANOVA), with group being a between-subject factor and hemisphere a within-subjects factor. To compensate for multiple comparisons and control the familywise Type I error rate at 5% in each family of four tests, we applied the Holm step-down criteria, setting the significance threshold for the strongest contrast at *p* = 0.05/4; for the second strongest at *p* = 0.05/3; and so forth (Bender and Lange, 2001). We calculated a Holm adjusted *p*-value as 4, 3, 2, or 1 times the observed value for the strongest, second strongest, and so forth. Normality assumption was tested with the Shapiro-Wilk test, sphericity assumption with the Mauchly test, and equality of variances with the Levene test. For the developmental trajectories of diffusion parameters, comparisons between hemispheres were performed by a linear mixed-effects model to account for within-subject correlations, with age, lesion and age × lesion interaction as fixed-effects. Comparisons between the two groups (HCP vs. TD) were also performed using a linear model. Linear regression analysis with a straight-line model was performed to test for a relationship between rMT and age. To test whether comparisons between slopes of the MA and LA hemispheres in children with HCP are significantly different, analysis of covariance (ANCOVA) was used. Since no TD children underwent motor mapping with TMS, only comparisons between the two hemispheres within the HCP group were performed for the rMT values. To avoid exclusion of patients with the highest rMTs, for those subjects (*n* = 3) whose APB rMT was >100% MO, the threshold for activation was estimated, per hemisphere, by sorting APB MEP peak-to-peak amplitudes, and obtaining the average e-field values (V/m) for the top 50th percentile. For all statistical analyses, the level of significance was set at *p* < 0.05.

## Results

### CST Diffusion Parameters for HCP and TD Children

ANOVA showed significant differences between the HCP and TD children for the FA (*F*_(1,64)_ = 9.860; *p* = 0.003), MD (*F*_(1,64)_ = 6.492; *p* = 0.013), AD (*F*_(1,64)_ = 6.553; *p* = 0.013), and RD (*F*_(1,64)_ = 7.578; *p* = 0.008; Figure 1). However, the main effect of hemisphere and the hemisphere × group interaction were not significant, indicating that the group difference was of similar magnitude in the MA and LA hemispheres.

### Maturational Trajectory of Diffusion Parameters

**Figure 2:**
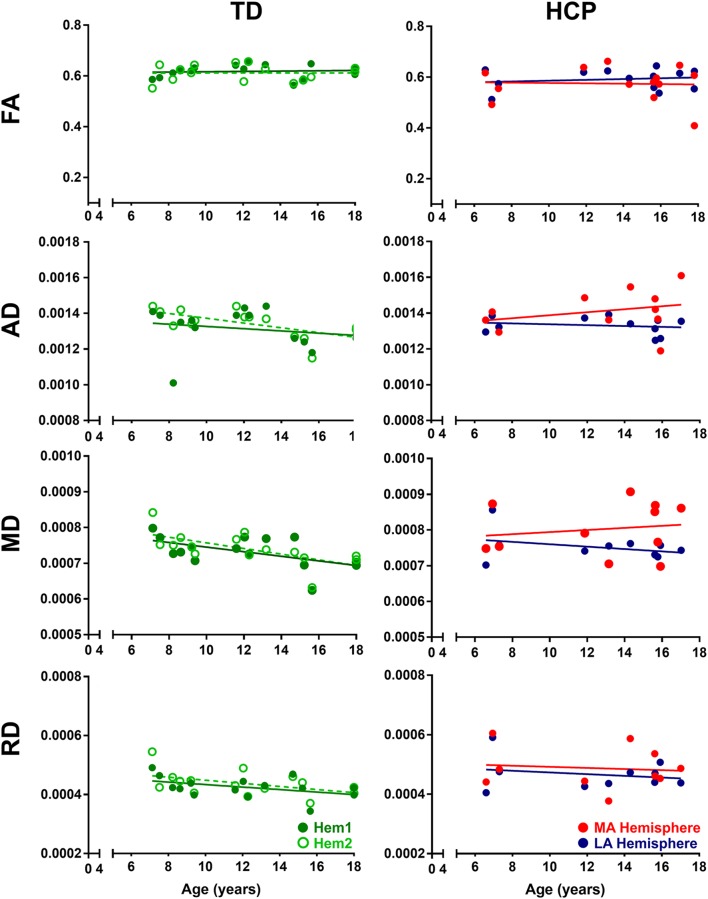
Developmental trajectories of diffusion parameters. Mean FA, AD, MD, and RD for TD children (Hem1: dark green closed circles, Hem2: light green open circles), and children with HCP (MA: red closed circles, LA: blue closed circles) as a function of age. For FA TD (Hem1; *R*^2^ = 0.0080, *p* = 0.7506), TD (Hem2; *R*^2^ = 0.0001, *p* = 0.9788), HCP (LA; *R*^2^ = 0.0258, *p* = 0.5999), HCP (MA; *R*^2^ = 0.0019, *p* = 0.8883). For AD: TD (Hem1; *R*^2^ = 0.4101, *p* < 0.001), TD (Hem2; *R*^2^ = 0.460, *p* < 0.001), HCP (LA; *R*^2^ = 0.0395; *p* = 0.558); HCP (MA; *R*^2^ = 0.081, *p* = 0.397). For MD: TD (Hem1; *R*^2^ = 0.299, *p* < 0.001), TD (Hem2; *R*^2^ = 0.419, *p* < 0.001), HCP (LA; *R*^2^ = 0.116, *p* = 0.306); HCP (MA; *R*^2^ = 0.025, *p* = 0.639). For RD: TD (Hem1; *R*^2^ = 0.1931, *p* = 0.101), TD (Hem2; *R*^2^ = 0.212, *p* = 0.085), HCP (LA; *R*^2^ = 0.0479, *p* = 0.5437); HCP (MA; *R*^2^ = 0.0117, *p* = 0.7665).

In TD children, there is a progressive decline with age for both AD and MD that is essentially identical in both hemispheres (Figure 2). This developmental trajectory is significant for both hemispheres of TD children (*p* < 0.05). FA and RD measures for Hem1 and Hem2 show absence of inter-hemispheric difference (Figure 1B), and neither FA nor RD change with age (*p* > 0.05, n.s.).

In contrast to TD children, the AD and MD parameters in children with HCP have no significant interaction with age in the MA (MD, AD: *p* > 0.05, n.s.) or LA (MD, AD: *p* > 0.05, n.s.) hemispheres. Though in line with findings for the TD children, neither FA nor RD values change with age in the HCP cohort (*p* > 0.05, n.s.; Table 2). The difference in slopes for TD Hem1/Hem2 vs. CP MA/LA is significant for both MD and AD diffusion parameters (*p* < 0.01).

**Table 2:** Maturational trajectory of diffusion parameters.

	AD	MD	FA	RD
TD (Hem1)	*R*^2^ = 0.4101*, *p* = 0.014	*R*^2^ = 0.2897*, *p* = 0.0385	*R*^2^ = 0.008045, *p* = 0.7506	*R*^2^ = 0.1932, *p* = 0.1012
TD (Hem2)	*R*^2^ = 0.460*, *p* = 0.008	*R*^2^ = 0.4099*, *p* = 0.0101	*R*^2^ = 0.00005658, *p* = 0.9788	*R*^2^ = 0.2118, *p* = 0.0843
HCP (MA)	*R*^2^ = 0.08095, *p* = 0.3965	*R*^2^ = 0.02547, *p* = 0.6393	*R*^2^ = 0.001873, *p* = 0.8883	*R*^2^ = 0.01167, *p* = 0.7665
HCP (LA)	*R*^2^ = 0.0395, *p* = 0.558	*R*^2^ = 0.1156, *p* = 0.3063	*R*^2^ = 0.02583, *p* = 0.5999	*R*^2^ = 0.04786, *p* = 0.5437

*p < 0.05.

### TMS Measure of CST Excitability and Maturation

**Figure 3:**
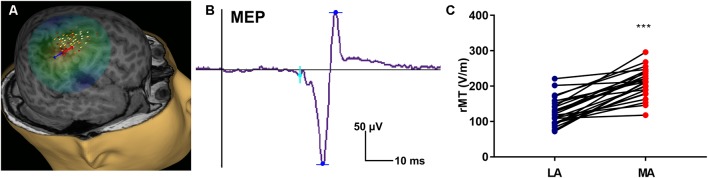
Representative transcranial magnetic stimulation (TMS) motor map and resting motor threshold (rMT) measures. **(A)** An approximation of stimulating electric field induced by TMS is displayed on a 3D reconstruction of the cortical surface (for same subject as shown in Figure 1A), where field center is indicated by the junction between the red and blue arrows indicating the direction of induced current, and corresponding composite map of right hemispheric stimulation sites evoking motor evoked potentials (MEPs) of the left abductor pollicis brevis (APB) muscle. Intensity of response is color-coded from lowest (gray) to highest (white). **(B)** Representative left APB MEP sample where the vertical line (black) corresponds to stimulus time. **(C)** rMT of children with HCP for the LA and MA hemispheres (***p < 0.0001).

**Figure 4:**
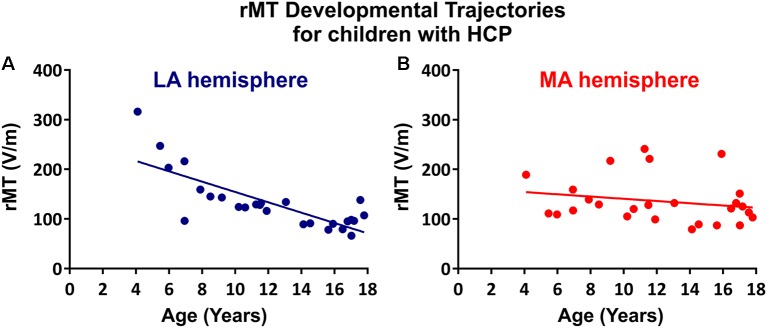
rMT developmental trajectories. rMT as a function of age in the **(A)** LA hemisphere (*R*^2^ = 0.6114, *p* < 0.0001) and **(B)** MA hemisphere (*R*^2^ = 0.0313, *p* = 0.4991) for patients with HCP who underwent TMS (*n* = 26).

The rMT was obtained per subject, per hemisphere for 26 subjects (Figure 3). Relevant to the present report, the rate of maturation differs between MA and LA hemispheres in patients with HCP. In the LA hemisphere, age is the major rMT determinant, which decreases by ~10.42 V/m per year throughout childhood (*R*^2^ = 0.605; *p* < 0.0001; Figure 4A). In contrast, rMT maturational trajectory is absent in the MA hemisphere (*R*^2^ = 0.012; *p* = 0.597; Figure 4B), indicating absent CST maturation specific to the MA hemisphere.

## Discussion

Through a multimodal neuroimaging approach, this cross-sectional study shows for the first-time evidence of disrupted CST maturation in both hemispheres of children with HCP. With DTI, we identified a diffusivity decrement (reduced AD and MD) with increasing age in both hemispheres of TD children. In contrast, we identified a diffusivity increment (increased AD and MD) in the MA hemisphere of children with HCP, and a steady diffusivity across age in the LA hemisphere. In complement to the DTI findings, by TMS, we also found a halted electrophysiological maturation of the CST in the MA hemisphere of children with HCP, which contrasts to a normal maturation in the LA hemisphere of children with HCP (Hameed et al., 2017; Säisänen et al., 2018). Our findings support our main hypothesis that perinatal injury arrests normal CST development. This arrest occurs in both hemispheres of children with HCP but is more pronounced in the MA hemisphere.

Numerous cross-sectional studies have investigated age-related differences in DTI parameters in healthy children and adolescents. These studies consistently demonstrate an increasing FA, a parameter linked to axon packing and myelination (Beaulieu, 2002), and a decreasing MD, a parameter reflecting water content and density, throughout brain white matter during childhood and adolescence (Schmithorst and Dardzinski, 2002; Eluvathingal et al., 2007; Mukherjee et al., 2008). Specifically, for the CST, Lebel and Beaulieu (2011) found a significant increase of the tract volume, decrease of FA, and increase of MD across ages 5–30 years. More recently, Yeo et al. (2014) observed a steep increase of FA until age 7 years and then a more gradual increase until adulthood, but did not examine other diffusion parameters, such as the AD, MD, or RD. As we have done, measuring all four diffusion parameters is important as each provides a distinct mechanistic insight into the HCP pathophysiology (Scheck et al., 2012), and each does not necessarily vary with age. For instance, while we identified a MD and AD reduction with increasing age we found no age-dependent FA change. Our findings are in line with previous DTI studies showing a maturation trajectory for the CST with decreasing diffusivity across age until the young adulthood. They are also consistent with the development of precise fine motor skills, which are dependent on CST maturation and continue to develop into young adulthood (Savion-Lemieux et al., 2009).

By DTI, we identified arrested maturation in both hemispheres of children with HCP as indicated by absent AD and MD change as a function of age (Figure 2). The arrest in DTI maturation was more prominent in the MA compared to the LA hemisphere. AD is specific to axonal degeneration (Song et al., 2005). Increased AD is associated with axonal injury or damage, which leads to reduced axonal density or caliber, or axonal loss, increasing the extra-axonal space by allowing faster water molecule movement parallel to axons (Song et al., 2005; Sun et al., 2008). The MD is a measure of intra- and extra-cellular water diffusion (Neil et al., 1998) and provides valuable information about diffusivity and myelination (Grant et al., 2001). Increased MD suggests increased extracellular water content due to gliosis and microscopic cystic changes that are reliable pathologic features of cerebral palsy. An increasing or steady AD and MD with increasing age for both hemispheres of children with HCP indicates an arrested maturation of the CST possibly as a result of disrupted myelination due to perinatal oligodendrocyte or oligodendrocyte progenitor injury (Volpe, 2009).

Our TMS findings further support lateralized CST developmental compromise in HCP. Notably, whereas CST excitability, possibly reflecting improving myelination, increases with age, we found that the developmental rMT trajectory was absent in the MA hemisphere in our subjects. In principle, absent age-dependent decline in rMT (corresponding to increasing CST excitability) may be interpreted as either delayed or accelerated maturation. That is, the developmental trajectory may be halted because development does not occur, or because development completes prematurely. In our case, the relatively low rMT in the MA hemisphere likely indicates a premature acceleration of CST excitability, corresponding to an early increase in CST excitability, and perhaps an early closure of the critical period for motor development in the injured hemisphere—this electrophysiologic finding may correspond to absent CST myelination that is identified by DTI. Interestingly, while DTI metrics indicate bilateral abnormalities in patients with HCP, TMS found that the maturation trajectory of the rMT was absent only in the MA hemisphere, which may indicate that DTI is a more sensitive instrument for detecting CST developmental compromise. Alternatively, preserved maturation in the contralesional hemisphere that is identified by TMS indicates compensatory cortical or spinal changes that enable normal maturation despite modest abnormalities in myelination or other microstructural elements that are indicated by DTI.

## Limitations

A limitation of this study is the lack of TMS data for TD children, and the fact that DTI and TMS data were not available for all participants with HCP given the risk (albeit small) of seizure or other adverse event associated with TMS, we could not justify administering this to healthy controls. Moreover, the participants with HCP had heterogeneities in the time, size, and location of the injury. Thus, the developmental changes observed in our cohort may not apply to all underlying pathologies of HCP. Finally, there are insufficient data to explain the differences in the changes of diffusion parameters. In particular, diffusion parameter differences may arise from a variety of factors, including differences in myelination, axonal fiber density and caliber, and fiber tract homogeneity, making it difficult to interpret the underlying pathology of the observed differences.

## Conclusion

We present evidence of disrupted CST maturation in both hemispheres of children with HCP possibly as a result of the perinatal injury using a multimodal neuroimaging approach. Despite its limitations, this cross-sectional study provides detailed insights into the neurophysiological mechanisms of development that follow a perinatal brain injury and may help monitoring the efficiency of interventions during critical periods of life.

## Data Availability

All datasets generated for this study are included in the manuscript.

## Ethics Statement

This study was carried out in accordance with the recommendations of Boston Children’s Hospital Internal Review Board (IRB). All subjects gave written informed consent in accordance with the Declaration of Helsinki. The protocol was approved by Boston Children’s Hospital IRB (IRB-P00023570; PI: CP).

## Author Contributions

CP, HK and AR contributed to the conception and design of the study. CP and HK contributed to the acquisition and analysis of data. CP and HK contributed to the drafting of figures. All authors contributed to the drafting of a significant portion of the manuscript.

## Conflict of Interest Statement

The authors declare that the research was conducted in the absence of any commercial or financial relationships that could be construed as a potential conflict of interest.

## Footnotes

^1^https://fsl.fmrib.ox.ac.uk/fsl/fslwiki/FDT

^2^http://surfer.nmr.mgh.harvard.edu/

^3^http://trackvis.org/

## Abbreviations

AD: Axial Diffusivity
ANOVA: Analysis of Variance
ANCOVA: Analysis of Covariance
APB: Abductor Pollicis Brevis
CC: Corpus Callosum
CE: Cystic Encephalomalacia
ChRIS: Children’s Research Integration System
CST: Corticospinal Tracts
Dom: Dominant
DTI: Diffusion Tensor Imaging
EMG: Electromyogram
EP: Echo-Planar
FA: Fractional Anisotropy
FACT: Fiber Assignment by Continuous Tracking
FOV: Field of view
GMFCS: Gross Motor Function Classification System
HCP: Hemiplegic Cerebral Palsy
GRAPPA: Generalized Autocalibrating Partial Parallel Acquisition
LA: Less Affected
MA: More Affected
MACS: Manual Abilities Classification Scale
MD: Mean Diffusivity
MEP: Motor Evoked Potential
MO: Machine Output
MPRAGE: Magnetization-Prepared Rapid-Acquisition Gradient-Echo
MRI: Magnetic Resonance Imaging
NDom: Non-dominant
PrG: Pre-Central Gyrus
PV-WMI: Periventricular White Matter Injury
RD: Radial Diffusivity
rMT: Resting Motor Threshold
ROI: Region of Interest
SMU: Sensorimotor U-fibers
STh: Spinothalamic
TD: Typically Developing
TE: Echo Time
ThC: Thalamacortical
TMS: Transcranial Magnetic Stimulation
TR: Repetition Time.

## References

[B1] Beaulieu C. (2002). The basis of anisotropic water diffusion in the nervous system—a technical review. NMR Biomed. 15, 435–455. 10.1002/nbm.782 12489094

[B100] Bender R. Lange S. (2001). Adjusting for multiple testing—when and how? J. Clin. Epidemiol. 54, 343–349. 11297884 10.1016/s0895-4356(00)00314-0

[B2] Chiappa K. H. Cros D. Day B. Fang J. J. Macdonell R. Mavroudakis N. (1991). Magnetic stimulation of the human motor cortex: ipsilateral and contralateral facilitation effects. Electroencephalogr. Clin. Neurophysiol. 43, 186–201. 1773757

[B3] Eluvathingal T. J. Hasan K. M. Kramer L. Fletcher J. M. Ewing-Cobbs L. (2007). Quantitative diffusion tensor tractography of association and projection fibers in normally developing children and adolescents. Cereb. Cortex 17, 2760–2768. 10.1093/cercor/bhm003 17307759 PMC2084482

[B4] Eyre J. A. Miller S. I. Clowry G. J. (2002). “The development of the corticospinal tract in humans,” in Handbook of Transcranial Magnetic Stimulation, eds Pascual-Leone A. Davey G. Rothwell J. Wasserman E. M. (London: Arnold), 235–249.

[B5] Eyre J. A. Miller S. Ramesh V. (1991). Constancy of central conduction delays during development in man: investigation of motor and somatosensory pathways. J. Physiol. 434, 441–452. 10.1113/jphysiol.1991.sp018479 2023125 PMC1181427

[B6] Eyre J. A. Taylor J. P. Villagra F. Smith M. Miller S. (2001). Evidence of activity-dependent withdrawal of corticospinal projections during human development. Neurology 57, 1543–1554. 10.1212/wnl.57.9.1543 11706088

[B7] Garvey M. A. Ziemann U. Bartko J. J. Denckla M. B. Barker C. A. Wassermann E. M. (2003). Cortical correlates of neuromotor development in healthy children. Clin. Neurophysiol. 114, 1662–1670. 10.1016/s1388-2457(03)00130-5 12948795

[B8] Grant P. E. He J. Halpern E. F. Wu O. Schaefer P. W. Schwamm L. H.. (2001). Frequency and clinical context of decreased apparent diffusion coefficient reversal in the human brain. Radiology 221, 43–50. 10.1148/radiol.2211001523 11568319

[B9] Hagberg B. Hagberg G. Beckung E. Uvebrant P. (2001). Changing panorama of cerebral palsy in Swede. VIII. Prevalence and origin in the birth year period 1991–94. Acta Paediatr. 90, 271–277. 10.1080/08035250117296 11332166

[B10] Hameed M. Q. Dhamne S. C. Gersner R. Kaye H. L. Oberman L. M. Pascual-Leone A.. (2017). Transcranial magnetic and direct current stimulation in children. Curr. Neurol. Neurosci. Rep. 17:11. 10.1007/s11910-017-0719-0 28229395 PMC5962296

[B12] Julkunen P. Säisänen L. Hukkanen T. Danner N. Könönen M. (2012). Does second-scale intertrial interval affect motor evoked potentials induced by single-pulse transcranial magnetic stimulation? Brain Stimul. 5, 526–532. 10.1016/j.brs.2011.07.006 21962979

[B13] Kaye H. L. Rotenberg A. (2017). “nTMS in pediatrics: special issues and solutions,” in Navigated Transcranial Magnetic Stimulation in Neurosurgery, ed. Krieg S. M. (New York, NY: Springer), 209–218.

[B14] Koh T. H. Eyre J. A. (1988). Maturation of corticospinal tracts assessed by electromagnetic stimulation of the motor cortex. Arch. Dis. Child. 63, 1347–1352. 10.1136/adc.63.11.1347 3202641 PMC1779172

[B15] Lebel C. Beaulieu C. (2011). Longitudinal development of human brain wiring continues from childhood into adulthood. J. Neurosci. 31, 10937–10947. 10.1523/JNEUROSCI.5302-10.2011 21795544 PMC6623097

[B16] Mori S. Crain B. J. Chacko V. P. van Zijl P. C. (1999). Three-dimensional tracking of axonal projections in the brain by magnetic resonance imaging. Ann. Neurol. 45, 265–269. 10.1002/1531-8249(199902)45:2<265::aid-ana21>3.0.co;2-3 9989633

[B17] Mukherjee P. Berman J. I. Chung S. W. Hess C. P. Henry R. G. (2008). Diffusion tensor MR imaging and fiber tractography: theoretic underpinnings. AJNR Am. J. Neuroradiol. 29, 632–641. 10.3174/ajnr.a1051 18339720 PMC7978191

[B18] Neil J. J. Shiran S. I. McKinstry R. C. Schefft G. L. Snyder A. Z. Almli C. R.. (1998). Normal brain in human newborns: apparent diffusion coefficient and diffusion anisotropy measured by using diffusion tensor MR imaging. Radiology 209, 57–66. 10.1148/radiology.209.1.9769812 9769812

[B19] Nezu A. Kimura S. Uehara S. Kobayashi T. Tanaka M. Saito K. (1997). Magnetic stimulation of motor cortex in children: maturity of corticospinal pathway and problem of clinical application. Brain Dev. 19, 176–180. 10.1016/s0387-7604(96)00552-9 9134188

[B20] Paus T. Collins D. L. Evans A. C. Leonard G. Pike B. Zijdenbos A. (2001). Maturation of white matter in the human brain: a review of magnetic resonance studies. Brain Res. Bull. 54, 255–266. 10.1016/s0361-9230(00)00434-2 11287130

[B21] Pihko E. Nevalainen P. Vaalto S. Laaksonen K. Mäenpää H. Valanne L.. (2014). Reactivity of sensorimotor oscillations is altered in children with hemiplegic cerebral palsy: a magnetoencephalographic study. Hum. Brain Mapp. 35, 4105–4117. 10.1002/hbm.22462 24522997 PMC6869593

[B22] Rosenbaum P. Paneth N. Leviton A. Goldstein M. Bax M. Damiano D.. (2007). A report: the definition and classification of cerebral palsy April 2006. Dev. Med. Child Neurol. 49, 8–14. 10.1111/j.1469-8749.2007.tb12610.x 17370477

[B23] Säisänen L. Julkunen P. Lakka T. Lindi V. Könönen M. Määttä S. (2018). Development of corticospinal motor excitability and cortical silent period from mid-childhood to adulthood - a navigated TMS study. Neurophysiol. Clin. 48, 65–75. 10.1016/j.neucli.2017.11.004 29274767

[B24] Savion-Lemieux T. Bailey J. A. Penhune V. B. (2009). Developmental contributions to motor sequence learning. Exp. Brain Res. 195, 293–306. 10.1007/s00221-009-1786-5 19363605

[B25] Scheck S. M. Boyd R. N. Rose S. E. (2012). New insights into the pathology of white matter tracts in cerebral palsy from diffusion magnetic resonance imaging: a systematic review. Dev. Med. Child Neurol. 54, 684–696. 10.1111/j.1469-8749.2012.04332.x 22646844

[B26] Schmithorst V. J. Dardzinski B. J. (2002). Automatic gradient preemphasis adjustment: a 15-minute journey to improved diffusion-weighted echo-planar imaging. Magn. Reson. Med. 47, 208–212. 10.1002/mrm.10022 11754462

[B27] Song S. K. Yoshino J. Le T. Q. Lin S. J. Sun S. W. Cross A. H.. (2005). Demyelination increases radial diffusivity in corpus callosum of mouse brain. Neuroimage 26, 132–140. 10.1016/j.neuroimage.2005.01.028 15862213

[B28] Sun S. W. Liang H. F. Cross A. H. Song S. K. (2008). Evolving Wallerian degeneration after transient retinal ischemia in mice characterized by diffusion tensor imaging. Neuroimage 40, 1–10. 10.1016/j.neuroimage.2007.11.049 18187343 PMC2276530

[B29] Volpe J. J. (2009). Brain injury in premature infants: a complex amalgam of destructive and developmental disturbances. Lancet Neurol. 8, 110–124. 10.1016/S1474-4422(08)70294-1 19081519 PMC2707149

[B30] Yeo S. S. Jang S. H. Son S. M. (2014). The different maturation of the corticospinal tract and corticoreticular pathway in normal brain development: diffusion tensor imaging study. Front. Hum. Neurosci. 8:573. 10.3389/fnhum.2014.00573 25309378 PMC4163649

